# In Vitro Calcification of Bioprosthetic Heart Valves: Method Validation by Comparative Heart Valve Calcification Testing

**DOI:** 10.1111/aor.70015

**Published:** 2025-10-21

**Authors:** Nicole Kiesendahl, Christoph Schmitz, Lars Peters, Marek Weiler, Thomas Schmitz‐Rode, Ulrich Steinseifer, Johanna C. Clauser

**Affiliations:** ^1^ Department of Cardiovascular Engineering, Institute of Applied Medical Engineering Medical Faculty RWTH Aachen University Aachen Germany; ^2^ ac.biomed GmbH Aachen Germany; ^3^ Institute of Crystallography RWTH Aachen University Aachen Germany; ^4^ Institute of Experimental Molecular Imaging RWTH Aachen University Aachen Germany; ^5^ Institute of Applied Medical Engineering Medical Faculty RWTH Aachen University Aachen Germany

**Keywords:** comparative calcification study, complexometric Ca quantification, histomorphologic localization of calcifications, hydroxyl apatite (HAP) quantification via μ‐CT, structural identification

## Abstract

**Background:**

A major reason for the failure of bioprosthetic heart valves is calcification. Various pretreatment methods are developed to reduce the calcification behavior. The effectiveness of these methods has so far been investigated in expensive and time‐consuming large animal studies. To provide a cost‐effective, animal‐ and possibly also time‐saving method, we developed an accelerated dynamic in vitro calcification test method.

**Methods:**

We validated this method using a comparative study of two differently pretreated groups of porcine heart valve bioprostheses. Each group contained *N* = 4 identical aortic bioprostheses. Calcification onsets, progression, and extent were detected by high‐speed video (HSV) documentation and microscopy. Structural identification of the deposits was carried out by X‐ray powder diffraction (XRD). Semi‐destructive quantification of the calcifications was done by μ‐CT as well as destructive chemical quantification via colorimetry and complexometry. The histomorphologic localization of the calcifications was examined by von Kossa staining.

**Results:**

Structural analysis of the deposits indicated “biological apatite” for both test groups. Histological examination revealed localization of the calcifications in the spongiosa zone of the cusps. Quantification of the calcifications showed a distinctly stronger calcification tendency of the No‐T6 compared to the anti‐calcifying pretreated T6 bioprostheses.

**Conclusions:**

We developed and validated a novel and unique test method for in vitro calcification assessment. The quantitative calcification tendencies of the two test groups are comparable with the results of an in vivo study in sheep. The structural findings are in line with published in vivo observations. The histomorphological localization appears as known for porcine prostheses.

## Graphical abstract

We have developed a unique method for pre‐clinical heart valve prostheses evaluation of calcification propensity. Results from the test can directly be translated into improvements of prostheses design, materials, and anti‐calcification treatments, and therefore have a significant impact on heart valve prostheses quality and patient care in the future.

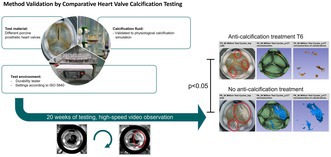

## 1 Introduction

Calcification is one major reason for the failure of bioprosthetic heart valves [1]. In (avital) bioprosthetic valve replacement, failure of the valves due to calcification‐related stiffening of the leaflets after about 10 to 12 years is a serious problem [1]. However, minimally invasive transcatheter aortic valve replacement (TAVR) has emerged as an alternative to traditional surgical valve replacement. Due to the rising numbers of TAVR, even in younger patients, and with regard to the increasing life expectancy of the elderly, the question of the durability of these prostheses becomes more important. However, durability analyses are mostly limited to a follow‐up period of eight years in most reports [2, 3, 4].

Usually, the calcification propensity of certain materials as well as potential anti‐calcification treatments is assessed in animal studies due to the lack of validated and standardized in vitro test options. Accordingly, calcification evaluation is only feasible in small cohorts and at a late stage of development. In order to facilitate dynamic in vitro heart valve calcification testing, we developed a unique accelerated in vitro calcification test method [5, 6]. Testing is based on a standard durability test according to ISO 5840 [7], using a special calcification fluid. In previous studies, we developed a fluid that prohibits spontaneous precipitation of calcific debris and is stable for at least one week. Changing the fluid weekly allows for calcification test periods of up to 20 weeks [5, 6].

The validation of this method by examining differently pretreated bioprosthetic heart valves concerning their calcification behavior in a comparative study is the last missing piece in the establishment of an in vitro calcification test method. In the present study, we validated our test method on two groups of bioprostheses, one with and one without anti‐calcification treatment. The focus here is not on comparing or evaluating the heart valve prostheses themselves, but on validating the test method. For this purpose, we deliberately chose two groups of heart valve prostheses whose calcification behavior is already known from animal studies [8], to validate in vitro with existing in vivo results. The calcification behavior of the prostheses was assessed by characterizing the calcification amount and characteristics. We compared various analytical methods (chemical and optical) to investigate the agreement, supplementation, or advantages and disadvantages of the different analytical methods.

## 2 Materials and Methods

The calcification propensity of two groups of prostheses (Hancock II Ultra T505 Size 25 mm, Medtronic, USA) differing in the pretreatment (standard T6 anti‐calcification treatment versus No‐T6 treatment) was tested in a comparative study under accelerated frequency and physiological pressure loads. Each group contained four identical bioprostheses.

### 2.1 Test System and Settings

The in vitro calcification assessment was conducted in a heart valve durability tester, CVE‐FT2 [5, 9]. The bioprostheses were clamped between customized ABS (acrylonitrile butadiene styrene) clamping rings (outer diameter: 38 mm, inner diameter: 26 mm) and mounted into separate test compartments by means of PVC (polyvinyl chloride) fixation nuts. All test compartments were filled with the same ionic calcification fluid described elsewhere [5, 6] at 37°C and a pH value of 7.4. The compartment lids were sealed with a flexible membrane to minimize fluid evaporation. Before assembly, the prostheses were immersed in 0.9% sodium chloride solution for 10 min at a temperature between 15°C and 25°C and afterwards rinsed twice with distilled water. Prostheses were loaded sinusoidally with a peak differential pressure across the closed valve of at least 100 mmHg consistent with normotensive conditions as specified for heart valve prostheses testing in the ISO 5840 [7]. Target peak differential pressure was maintained at all test cycles for 5% or more of the duration of each test cycle. The test frequency was 300 bpm. The test duration was 20 weeks or until prostheses' failure, respectively, to allow for comparison with previous in vitro and in vivo studies [6, 8]. The calcification fluid was changed weekly and analyzed in terms of the ionic concentrations.

### 2.2 Calcification Solution

The composition of the barbital buffered calcification solution is based on Fluid L of the preliminary fluid study [6] with a slight decrease in the total calcium concentration Ca_T_. The used fluid still had an ionic strength (I) of 0.16 M [5, 6, 10, 11] as assumed for blood plasma, and was still undersaturated with respect to the thermodynamic solubility constant K_Sp_ of dicalciumphosphate‐dihydrate (DCPD). Table 1 shows the exact composition of the fluid. The fluid preparation and preservation were performed as previously described [5, 6].

**TABLE 1:** Fluid composition of the used fluid, based on fluid L from Kiesendahl et al. [6].

CaCl_2_	1.8 mM
Ca_T_	1.8 × 10^−3^ M
KH_2_PO_4_	1.0 mM
P_T_	1.0 × 10^−3^ M
NaCl	115 mM
KCl	4.0 mM

## 3 Analysis

### 3.1 Fluid Composition

The initial concentrations of total calcium and total phosphate in the fluid were determined by colorimetry as well as the calcium and phosphate concentrations within each compartment over the test duration, as described earlier [6].

### 3.2 Light Microscopy and High‐Speed Video Documentation

The localization and extent of calcification as well as deposits and possible defects in the valve material were assessed by comparative light microscopy prior to testing and after test completion or prostheses' failure, respectively. The calcification propensity and progression during the test were detected macroscopically and by high‐speed video (HSV) monitoring using a Photron Fastcam Ax 50 with a Nikkor AF Micro 60 mm lens on the prostheses' leaflets.

### 3.3 μ‐CT

For 3‐dimensional imaging and semi‐destructive quantification of the calcifications of each whole valve as hydroxyapatite (HAP), μ‐CT (micro Computed Tomography) was conducted after completion of the test with a CT with Optical Imaging (CT‐OI) based on Vector 4 CT/UHR μ‐CT scanner (Milabs B.V., Houten, The Netherlands). The quantification of the calcifications as mass of HAP/prosthesis was determined by converting the obtained volume scores (V_Score_) and mean CT values (CT) according to the following relation (Equation 1):

(1) $$m\;\left(\mathrm{HAP}\right)=\rho \;\left(\mathrm{HAP}\right)\times V=c_{\mathrm{HAP}}\times \mathrm{CT}\times V_{\text{Score}}\;\left(c_{\mathrm{HAP}}=\text{calibration factor}\right)$$

by using a HAP calibration phantom (Phantom QSA 212) with five different HAP‐concentration inserts [12]. Since the stent and radiopaque stent markers interfere with μ‐CT scans due to artifacts, all prostheses were dissected from the stents before μ‐CT.

### 3.4 Chemical Quantification by Colorimetry and Complexometry

Chemical quantification of calcium and phosphate was performed colorimetrically and complexometrically after deposits were extracted from the leaflets. In the case of visually distinct deposits, the halves of each of the three leaflets of the prosthesis were systematically selected. In the case of only slight visual calcifications, all three leaflets were used completely. A detailed description of the preparation process is available in the Supporting Information.

### 3.5 X‐Ray Powder Diffraction (XRD)

Structural analysis of deposits was carried out by X‐ray powder diffraction (Bruker AXS D8 Advance) between 10° and 100° 2θ in steps of 0.0105° with a measuring time of 0.5 s/step with Ni‐filtered Cu *Kα*_1,2_—radiation using a *LynxExe* semiconductor strip detector. Therefore, two calcified leaflets of each valve were cut in half and the deposits from each half were collected, dried as described elsewhere [6] and prepared onto flat silicone (Elastosil 911, Wacker AG, Germany) sample holders.

### 3.6 Histology

Histological examinations of calcified leaflet cross sections were made from 5 μm thin slices by Hematoxylin and Eosin (HE), Elastica van Gieson (EvG), and von Kossa staining. Details on the staining protocols are included in the Supporting Information.

## 4 Results

Over the course of 20 test weeks, the prostheses underwent 60 million test cycles; however, some of the prostheses had to be removed from the test prematurely due to calcification‐related leaflet defects. All prostheses showed signs of calcification during the test period, which differed in their onset and optical extent. The prostheses each had two larger and one smaller leaflet. The calcification onsets were located close to the suture ring in all prostheses, with one or both of the larger leaflets initially affected in seven of eight cases. During the test, calcifications spread to the leaflet planes to varying degrees.

### 4.1 High‐Speed Video Documentation

The high‐speed video documentation includes the analysis of the calcification onset, calcification progress, and localization. It allows the detection of tissue damages and prostheses failures, as well as the ongoing calcification progress (Figures S2, Supporting Information).

In the standard group with T6 calcification‐mitigation treatment, sample P4 showed the first sign of calcification with an onset at 18 million cycles (6 weeks); the other three prostheses in this group showed calcification onset at 33 million test cycles (11 weeks, P2) and 45 million test cycles (15 weeks, P1 and P3), respectively. At test termination (60 million cycles, 20 weeks), P1 showed clear calcification in the center of one of the two larger leaflets; the other two samples, P2 and P3, were still in the early stages of calcification. Sample P4 suffered so severely from calcification‐related hole formation that it had to be removed from the test at 36 million test cycles.

The very first visible signs of calcification were detected in the No‐T6 group at 9 million test cycles (3 weeks, P6) (Figure S2) and 15 million test cycles (5 weeks, P5 and P7) (Figure S2). Sample P8 showed the first sign of calcification at 24 million test cycles (8 weeks). All prostheses in this test group showed distinct calcification at the end of the test, whereby only P5 and P8 reached the 60 million cycles (20 weeks), while P6 and P7 had to be removed from the test after 36 million (12 weeks) and 45 million test cycles (15 weeks), respectively, due to calcification‐related hole formation.

### 4.2 Light Microscopy

**FIGURE 1:**
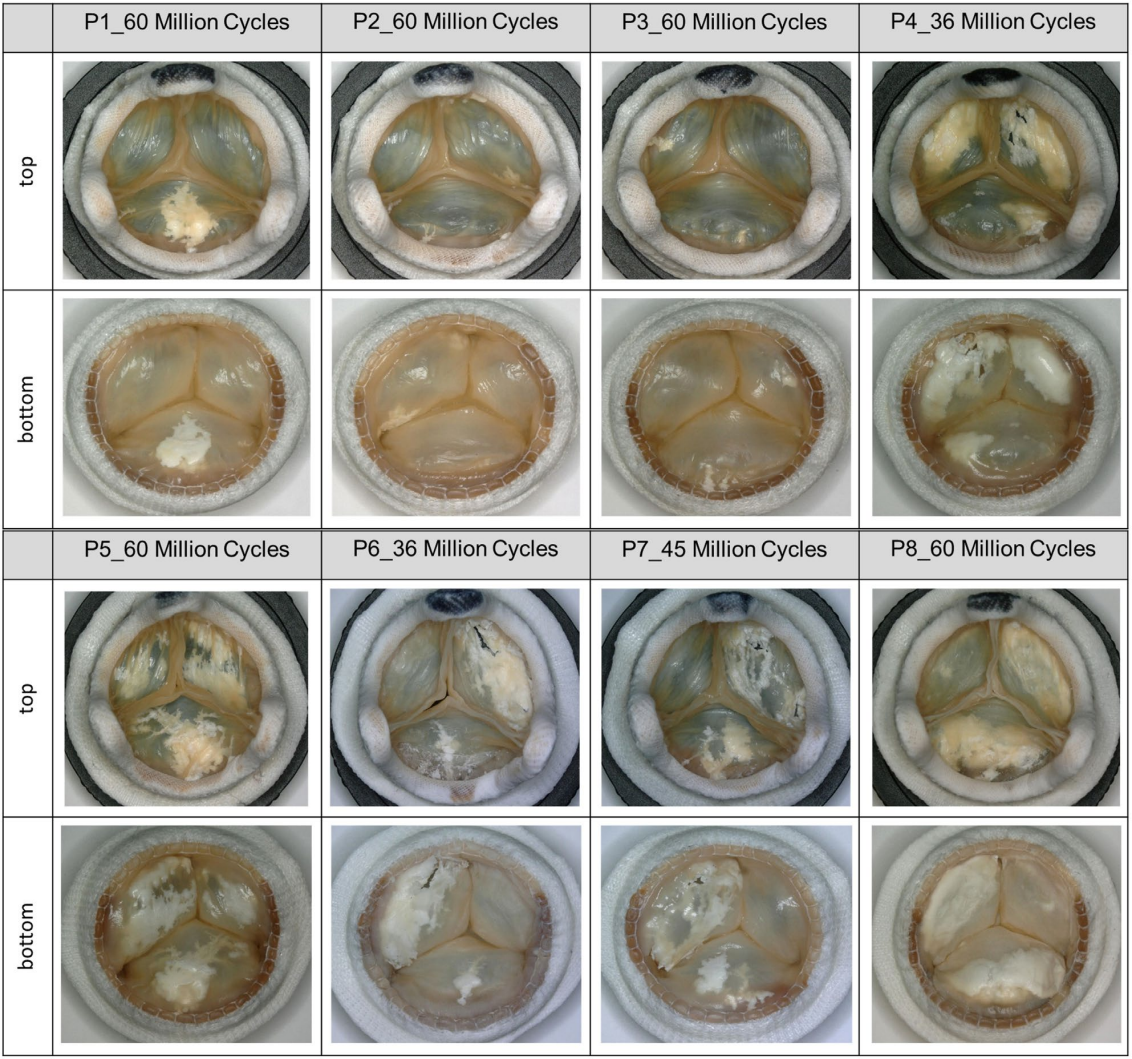
Overview of the optical calcification status of all examined test prostheses. Top and bottom sides of T6 group (P1–P4) and No‐T6 group (P5–P8) at the end of testing or at valve removal. [Color figure can be viewed at wileyonlinelibrary.com]

The optical calcification status of the top and bottom sides of all heart valves at the end of testing or at valve removal, respectively, showed that the prostheses of the No‐T6 group (P5–P8) reveal a stronger visual calcification than the samples P1–P3 of the standard T6 group (Figure 1). On microscopic examination, P4 seemed to be an exception in the standard T6 group due to the early severe calcification. In all prostheses, both the top and the bottom of the leaflets were affected by calcification.

**FIGURE 2:**
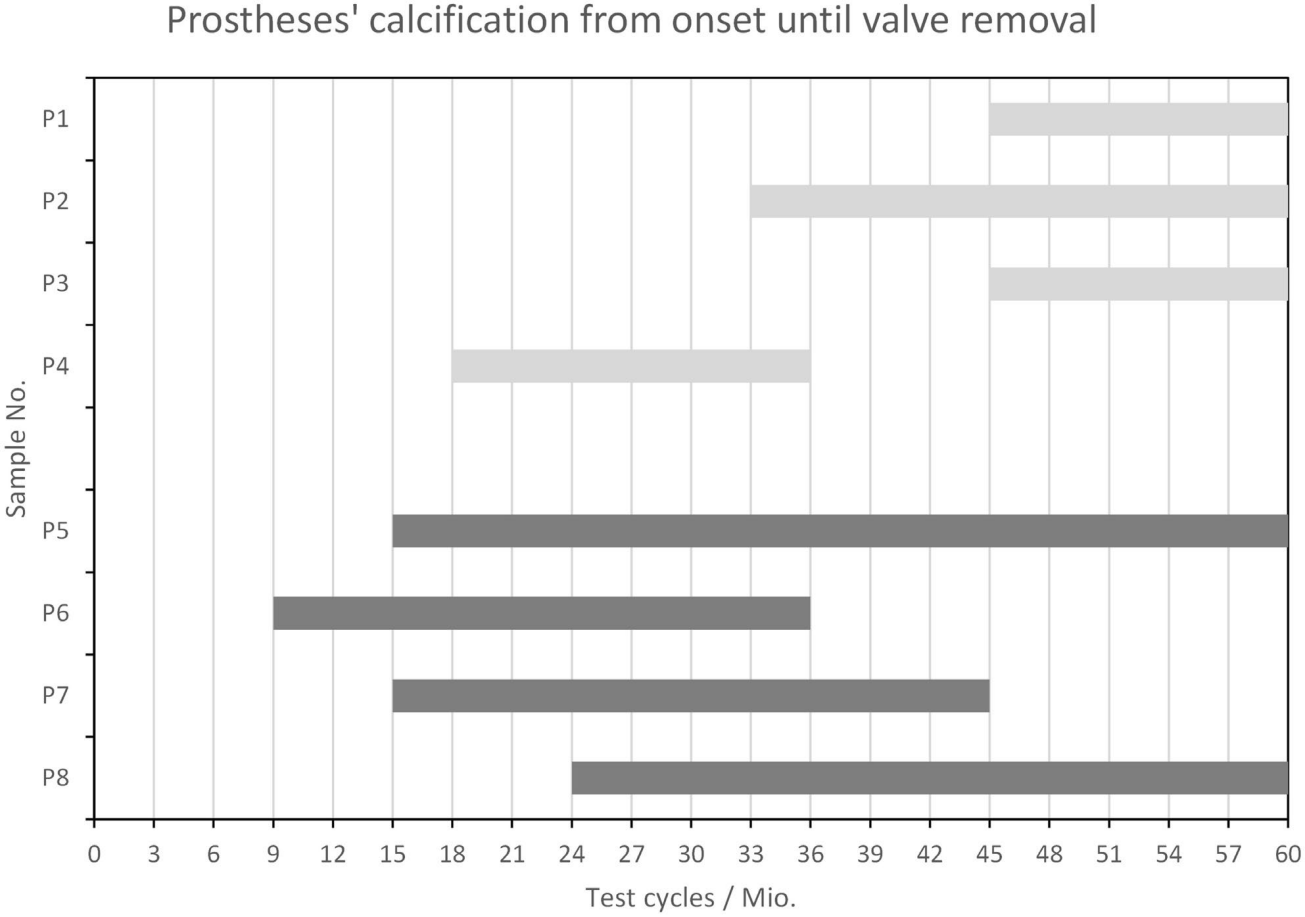
Overview of calcification onsets to valve removals for the two groups of prostheses.

Especially, if the test‐duration‐based calcification onsets and durations of valve survivals are taken into account (Figures S1 and S2), the prostheses of the T6 group (except for sample P4) showed better performance (Figure 2). From Figure 2 it is evident that in the standard T6 group, P4 deviated markedly from the behavior of the other three prostheses.

### 4.3 μ‐CT and Quantification of Calcifications as HAP

**FIGURE 3:**
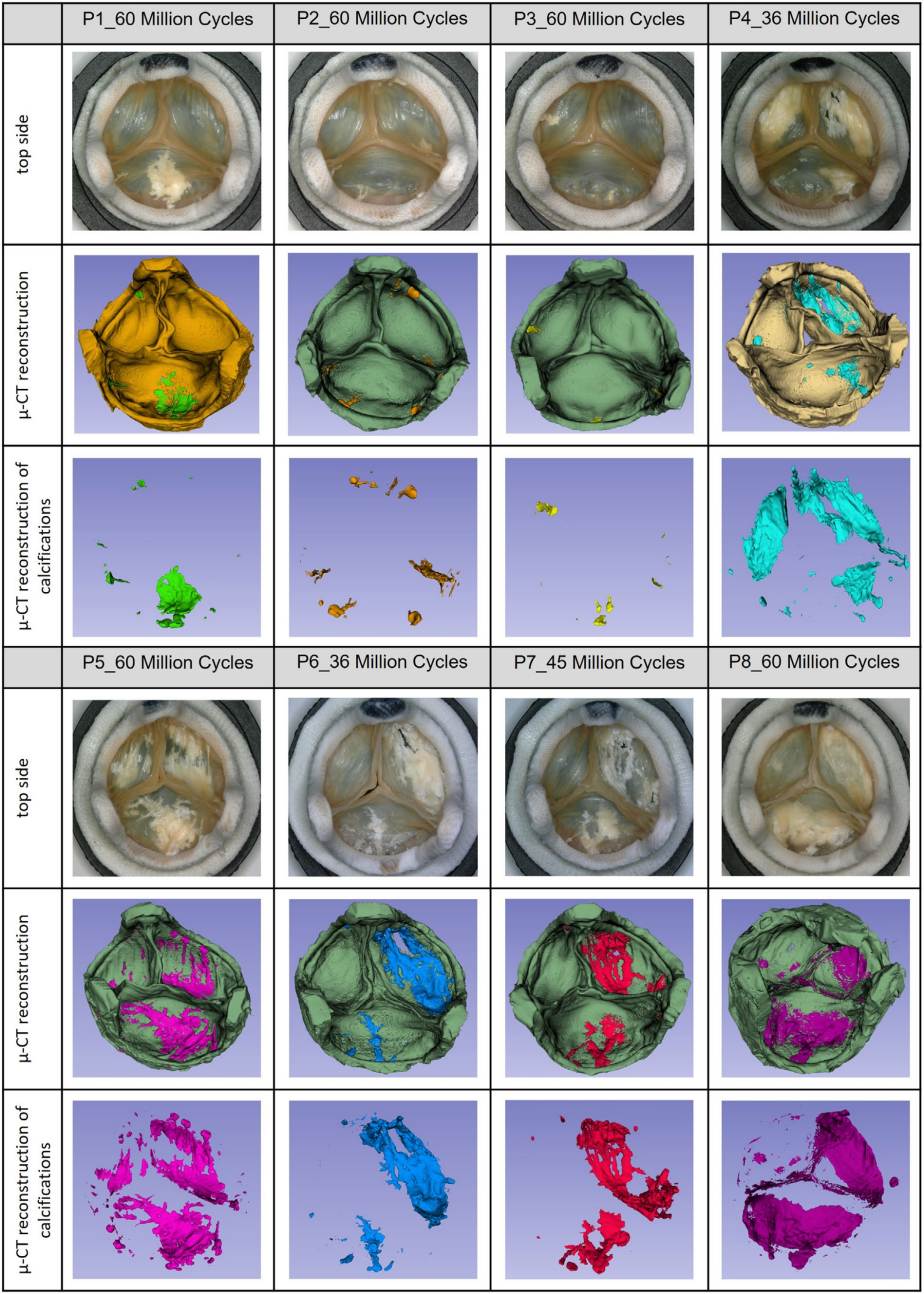
Comparison of microscopy images and μ‐CT reconstructions of the test samples. Reconstruction of the whole calcified prostheses and the calcifications separately (P1–P4 Standard T6 group, P5–P8 No‐T6 group). [Color figure can be viewed at wileyonlinelibrary.com]

The μ‐CT data confirms the results from the evaluation of the high‐speed video (Figures S1 and S2) and microscopic documentations (Figure 1). The No‐T6 prostheses (P5–P8) showed a stronger tendency for calcification than the standard T6 prostheses (P1–P4), whereby P4 is probably to be considered an outlier (Table 2 and Figure 3).

**TABLE 2:** Quantification of calcification as mass of HAP per prosthesis and stoichiometrically calculated Ca masses per prosthesis from the HAP data.

Test prosthesis		m (HAP)/prosthesis	m (Ca^2+^)/prosthesis (calculated from HAP mass)
Standard T6 group	P1	21.59 mg	8.60 mg
	P2	18.30 mg	7.29 mg
	P3	2.85 mg	1.13 mg
	P4	82.10 mg	32.69 mg
No‐T6 group	P5	96.92 mg	38.59 mg
	P6	69.34 mg	27.61 mg
	P7	93.30 mg	37.15 mg
	P8	89.72 mg	35.72 mg

Comparison of the μ‐CT reconstructions with the microscope images showed that calcifications located inside the tissue are still visible in the microscope images but are partially covered by leaflet tissue when the whole valve is segmented (Figure 3). They only become fully visible when the calcifications are segmented separately.

### 4.4 Chemical Quantification of Calcium and Phosphate

A detailed evaluation of the colorimetric analysis of calcium and phosphate and the complexometric calcium determination with regard to different calcium phosphate phases [13] as well as the normalization of the calcium masses obtained per test material to “calcium mass”/“dry tissue weight” is available in Tables S1–S4 in the Supporting Information.

A comparison of the calcium determination of both analysis methods by “calcium mass”/“tissue dry weight” is given in Table 3.

**TABLE 3:** Comparison of the colorimetrically and complexometrically determined Ca masses per g tissue dry weight.

Test prosthesis		Colorimetry m (Ca)/g tissue dry weight	Complexometry m (Ca)/g tissue dry weight
Standard T6 group	P1	15.03 mg	17.64 mg
	P2	16.51 mg	17.28 mg
	P3	2.22 mg	2.66 mg
	P4	120.05 mg	124.68 mg
No‐T6 group	P5	61.97 mg	67.14 mg
	P6	113.35 mg	108.35 mg
	P7	138.95 mg	133.45 mg
	P8	121.75 mg	124.36 mg

### 4.5 Structural Calcification Investigation via XRD

For the crystallographic investigation of calcifications, distinctly calcified prostheses from each group were selected as a representative sample. From the standard T6 group, prosthesis P4 was considered. Although it appears as an outlier in this group in terms of the extent of calcification, it exhibited sufficient calcified material to isolate the deposits from the valve tissue for this very reason. From the No‐T6 group, prosthesis P5 was selected because two leaflets of this valve were distinctly calcified. The measured diffractograms of the deposits of these two valves are comparable and shown in Figure S3 (Supporting Information).

### 4.6 Histology

**FIGURE 4:**
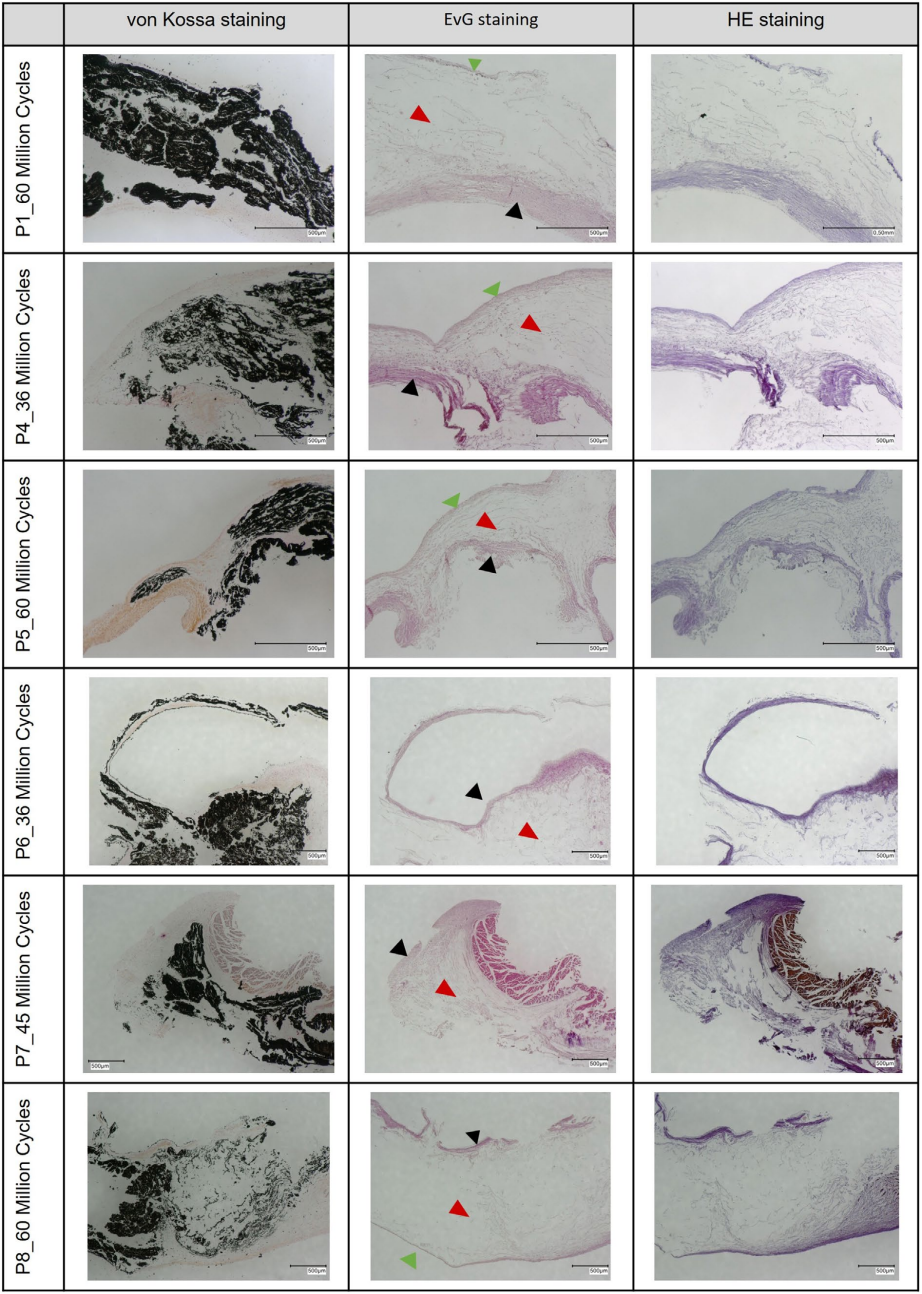
Histological staining (von Kossa, HE, and EvG) of leaflet cross sections of prostheses P1 and P4–P8. Black arrows showing the zona fibrosa, red arrows showing the spongiosa, and green arrows showing the ventricularis. Scale bars present 500 μm. [Color figure can be viewed at wileyonlinelibrary.com]

The histological examination of the test samples P1 and P4–P8 (Figure 4) showed in the von Kossa staining of the leaflet cross sections that calcifications are mainly localized in a sandwich‐like manner inside the leaflet tissue, as well as partly running along the leaflet surfaces. Comparison with HE and EvG staining shows that the calcifications are mainly localized in the spongiosa [14, 15], a loose connective tissue layer between fibrosa and ventricularis [16, 17].

In the calcified areas, the spongiosa appears distinctly scattered and widened, as if the calcification lies in a pocket. A similar picture of the arrangement of calcification between tissue layers, as in a pocket, had also been seen in the microscope‐guided leaflet dissection of samples P1, P4, and P5, and is exemplary compared to histology for P1 in Figure S4 (Supporting Information). On samples P2 and P3, no histological examinations were performed, as all the tissue was used for quantitative analysis.

### 4.7 Statistics

The quantification of the in vitro generated deposits as “HAP mass”/prosthesis or “calcium mass”/prosthesis from the μ‐CT data showed a significant difference between the mean values of the two test groups (Table 2). The statistical significance of the group differences was tested using an unpaired *t*‐test. The data is summarized in Tables S5 and S6 (Supporting Information).

The mean values of the quantifications as “calcium mass”/“tissue dry weight” from the colorimetric and complexometric analysis show the same trend for the two test groups as the μ‐CT data, but do not show statistical significance. In the T6 group, the data were not normally distributed as assessed by the Shapiro–Wilk test (*p* < 0.05). Regardless of this, a *t*‐test was performed. The data are summarized in Tables S7 and S8.

## 5 Discussion

We developed an accelerated in vitro calcification test method to provide a cost‐ and animal study‐saving test procedure for bioprosthetic heart valves. In a previous study, we addressed the problem of superficial calcification and spontaneous precipitation of so far applied fluids and developed a nearly physiological non‐spontaneous precipitating fluid [5]. To validate our test method, we herein conducted a comparative study of two groups of differently pretreated heart valve bioprostheses with known calcification behavior from animal study and literature [8], and evaluated different analyses for suitability. Our assessment included the initiation, progression, macroscopic localization and extent, structural appearance and crystallographic phase identification, quantification and the tissue morphological localization of the calcifications as well as a comparison with literature data [5, 8, 14, 18, 19].

### 5.1 Comparison of Quantification Methods

The μ‐CT reconstructions (Figure 3) and quantification data (Table 2), as well as the quantification by colorimetry and complexometry (Table 3), confirm the results from the evaluation of the high‐speed video and microscopic documentations (Figures S1, S2 and Figure 1). All methods show the same trend of calcification tendency with respect to the different pre‐treatments and thus confirm the calcification mitigating effect of the T6 pre‐treatment.

The advantage of quantification by μ‐CT is the only semi‐destructive preparation of the valve material, so that the tissue and calcification remain almost unaffected and are available for further examination. Regarding the quantitative calcium determination, complexometry appears superior to colorimetry with respect to the lower detection limit for minor calcifications. In general, the chemical quantifications are subject to more uncertainties due to the sample preparation (compare Supporting Information, p 10 ff). As the leaflets are usually not evenly calcified, the sample selection is not entirely representative. The splitting of the leaflets can be accompanied by a loss of material, and the subsequent acid digestion can lead to tissue loss, which may be reflected in variability between the calculated dry weight of the demineralized tissue and effective redrying mass.

For future calcification studies, it can be concluded that quantification via μ‐CT is the method of choice for studies in which, in addition to calcium quantification, further examinations such as XRD and histology are desired. If, on the other hand, only the pure calcium determination is required, complexometry appears to be the most suitable method.

### 5.2 Structural Deposit Analysis by XRD

Biological calcification can occur in different calcium phosphate phases. The “biological apatite” is not stoichiometrically pure hydroxyapatite but calcium‐deficient hydroxyapatite (CDHA). However, there is no own structure determination of the CDHA; it corresponds to the diffractogram of HAP [13]. Comparison of the measured diffractograms (Figure S3) with the reference maps for DCPD, OCP, and HAP [5] and the literature diffractograms of synthetic apatite and the deposits on a human aortic valve [18] as well as explanted porcine xenografts [15] showed that HAP (resp. CDHA) is the main phase of calcification. Slight differences in the lattice parameters of the apatites in both samples may indicate slightly varying overall compositions of the apatite (e.g., water content, defects) and underline the presence of “biological apatite” (Supporting Information).

### 5.3 Histology

The microstructure of native aortic human and porcine valve cusps shows a characteristic trilaminar architecture of distinct supposed layers: fibrosa, spongiosa, and ventricularis [19, 20], where the outer surfaces are covered by a monolayer of endothelial cells [16, 17, 19, 20]. When porcine aortic valves are processed to bioprostheses, they are usually subjected to glutaraldehyde stabilization, removing the endothelial cells partially or completely from the outer surfaces of the cusps. However, deadened cells or their fragments remain in the connective tissue layers, and the proteoglycan and glycosaminoglycan content is reduced, particularly in the spongiosa layer [15, 19]. This may represent a serious modification since some of these molecular complexes have been found to be calcification inhibitors [15, 19] and, in addition, dead cells may act as calcification nucleators due to their phosphate groups of lipid membranes [13, 21, 22]. Due to the reduced proteoglycan and glycosaminoglycan content, the spongiosa would also no longer be fully functional as a sliding layer, and the heart valves would be exposed to increased mechanical stress without protection, which would also increase the risk of valve calcification [15, 19, 20].

Both prostheses groups were glutaraldehyde‐fixed and subjected to mechanical stress during testing, which would suggest localization of calcification particularly in the spongiosa layer [15, 19]. In addition, collagen‐rich tissue, such as the fibrosa, is known to be prone to calcification [15, 19]. Nevertheless, the T6 pretreated prostheses exhibited significantly less calcification than the No‐T6 group (Tables S5 and S6), which suggests that the T6 treatment (surfactant treatment with sodium dodecyl sulfate) removes the phospholipids as calcification nucleators from the tissue and thus mitigates the calcification propensity [23].

When calcification did occur in the T6‐treated prostheses with a significant delay and/or mitigation, it was similarly localized in the tissue as in the No‐T6 prostheses (Figures 4 and S4), indicating the influence of the spongiosa affected by the processing and the mechanical stress on the valves on the calcification process [15, 19, 20].

### 5.4 Comparison of In Vitro Data With In Vivo Sheep Data

In an animal study by Jones et al., 28 Standard Hancock valves (No‐T6) and 17 pre‐treated Hancock II valves (T6) were implanted in the mitral position of juvenile sheep for 20 weeks [8]. The differences between the two test groups showed significantly less calcification of the T6‐treated prostheses compared to the No‐T6 prostheses, based on the calcium quantification per g tissue dry weight (Table S9) [8]. In our in vitro study, such significance was shown by quantification via μ‐CT as HAP/“whole prosthesis” or “calcium mass”/“whole prosthesis” calculated from HAP (Tables S5 and S6). Our quantifications as “Ca mass”/“tissue dry weight” also showed the trend of calcification mitigation of the T6 group compared to the No‐T6 group (Table 3), but did not show the statistical significance as in the quantification via μ‐CT or as in the animal study. This may be due to the uncertainties by leaflet division, acid treatment for dissolving the calcifications, and tissue drying, which are required for the chemical calcium determinations. In addition, the smaller number of cases in the in vitro study (*N* = 4) for each group compared to *N* = 17 or *N* = 28 for the in vivo study might reduce statistical significance.

Apart from the quantification of calcification, the in vitro study using HSV monitoring can also include the temporal progression of calcification from onset to valve removal (Figure 2) in the evaluation, which was not the case in vivo. This time course of calcification determined in vitro also confirms the calcification‐mitigating effect of the T6 treatment and, together with the quantification results, underlines the validity of our test method.

The test method validated in this study cannot be regarded as a complete substitute for testing systemically acting anti‐calcification treatments due to the simplified fluid composition to the essential physiological electrolyte ratios. A more comprehensive approach would require a systemic environment based on cell culture medium with the addition of proteins, enzymes, and possibly suitable cells, which is the subject of our ongoing research [6]. Furthermore, the X‐ray diffractometric investigation of the calcification structure was only possible on a random basis due to the limited amount of test material.

## 6 Conclusion

We developed and validated a novel test method for in vitro calcification assessment of prosthetic heart valves. After 20 weeks of testing, the results of the comparative study showed a distinct difference in calcification tendency between the two test groups, both in quantitative extent and in time course. The not‐pretreated prostheses (No‐T6) showed a greater and earlier tendency to calcify than the anti‐calcifying pretreated prostheses. The developed in vitro test method allowed the formation of calcifications comparable to in vivo calcifications in terms of crystal structure and histomorphologic localization in porcine prosthetic heart valves [5, 13, 14, 15, 18]. Besides the cost savings and the identical test conditions for up to 12 prostheses, the observability of the calcification progression by means of high‐speed video recordings also presents an advantage of the in vitro methodology. In this way, calcification starting areas could be localized. Regarding the quantitative calcium determination, complexometry appears superior to colorimetry. The advantage of quantification by μ‐CT is the only semi‐destructive preparation of the valve material.

In further studies, it would be interesting to go into more detail on the formation of the calcification starting points, e.g., suture insertion points, and to investigate the correlation to mechanical load. Not least, the test method developed and validated is also intended to provide a differentiated comparison of different anti‐calcification pretreatments.

## Author Contributions

N.K.: Concept, data collection, data analysis, statistics, interpretation and drafting of the article; C.S.: data collection; L.P.: data collection; M.W.: data collection; T.S.‐R. and U.S.: critical revision of the article; J.C.C.: interpretation, critical revision of the article.

## Conflicts of Interest

The authors declare no conflicts of interest.

## Supporting information


**Data S1:** Supporting Information.
