# Supplementary material for: In Vitro Calcification of Bioprosthetic Heart Valves: Method Validation by Comparative Heart Valve Calcification Testing
Source: Artif Organs. 2025 Oct 21;50(1):64–73. doi: 10.1111/aor.70015 (PMC12954470; doi:10.1111/aor.70015)
Supplement: Supplementary file 1 — Data S1: Supporting Information. [file AOR-50-64-s001.pdf]

<u>Supporting Information:</u> ***In-vitro Calcification of Bioprosthetic Heart Valves: Method Validation by Comparative Heart Valve Calcification Testing***

N Kiesendahl, C Schmitz, L Peters, M Weiler, T Schmitz-Rode, U Steinseifer, JC Clauser

Histological Staining

The histological stainings were performed according to the standard protocols of the Immunohistochemistry Facility of the Interdisciplinary Center for Clinical Research (IZKF) Aachen within the Faculty of Medicine at RWTH Aachen University.

Briefly, Hematoxylin and Eosin (HE) staining was performed using hematoxylin acid (Morphisto GmbH, Germany), rinsing in water, staining with eosin Y (0.5 %, aqueous, Morphisto GmbH, Germany). After rinsing with distilled water, samples were passed through a dehydration series before embedding for microscopy.

Elastica van Gieson (EvG) started with Weigert's resorcin-fuchsin solution (Carl Roth GmbH & Co. KG, Germany), followed by rinsing under fluent tap water and distilled water. After differentiation with 80 % ethanol, nuclei were stained with hematoxylin solutions A and B (Carl Roth GmbH & Co. KG, Germany), samples were rinsed and stained with van Gieson solution (Carl Roth GmbH & Co. KG, Germany) prior to dehydration and embedding.

Van Kossa staining started with 1% aqueous silver nitrate solution (Merck KGaA, Germany), following incubation in soda-formol, rinsing under fluent distilled water and incubation in 5 % sodium thiosulfate pentahydrate (Merck KGaA, Germany). After another rinsing interval with distilled water, nuclei staining was performed using a 0.1 % nuclear fast red–aluminum sulfate solution (Carl Roth GmbH & Co. KG, Germany) followed by rinsing with distilled water. Afterwards, samples were dehydrated and embedded as described before.

High-Speed Video Documentation – T6 treated prostheses

In the standard group with T6 calcification-mitigation treatment, sample P4 showed the first sign of calcification with an onset at 18 million cycles (6 weeks) near the suture ring of one of the larger leaflets (Fig. S1). The other three prostheses in this group showed calcification onset at 33 million test cycles / 11 weeks (P2) and 45 million test cycles / 15 weeks (P1 and P3), respectively, where the calcification onset at P3 was on the smaller leaflet (Fig. S1). At test termination (60 million cycles, 20 weeks), P1 showed clear calcification in the center of one of the two larger leaflets; no calcification was detected on the other two leaflets of this valve from the high-speed video documentations. The other two test samples P2 and P3 were still in the early stages of calcification in terms of their optical extent at the end of the test. P4 showed a moderate increase in calcification from week 6 to week 11, which changed during week

Corresponding author: Johanna C. Clauser; clauser@ame.rwth-aachen.de

11 into a massive large-scale calcification of one larger and the smaller leaflet with formation of a hole in the calcified area of the larger leaflet. During week 12, the test prosthesis suffered so severely from calcification-related hole-formation in the larger leaflet that it had to be removed from the test at 36 million test cycles.

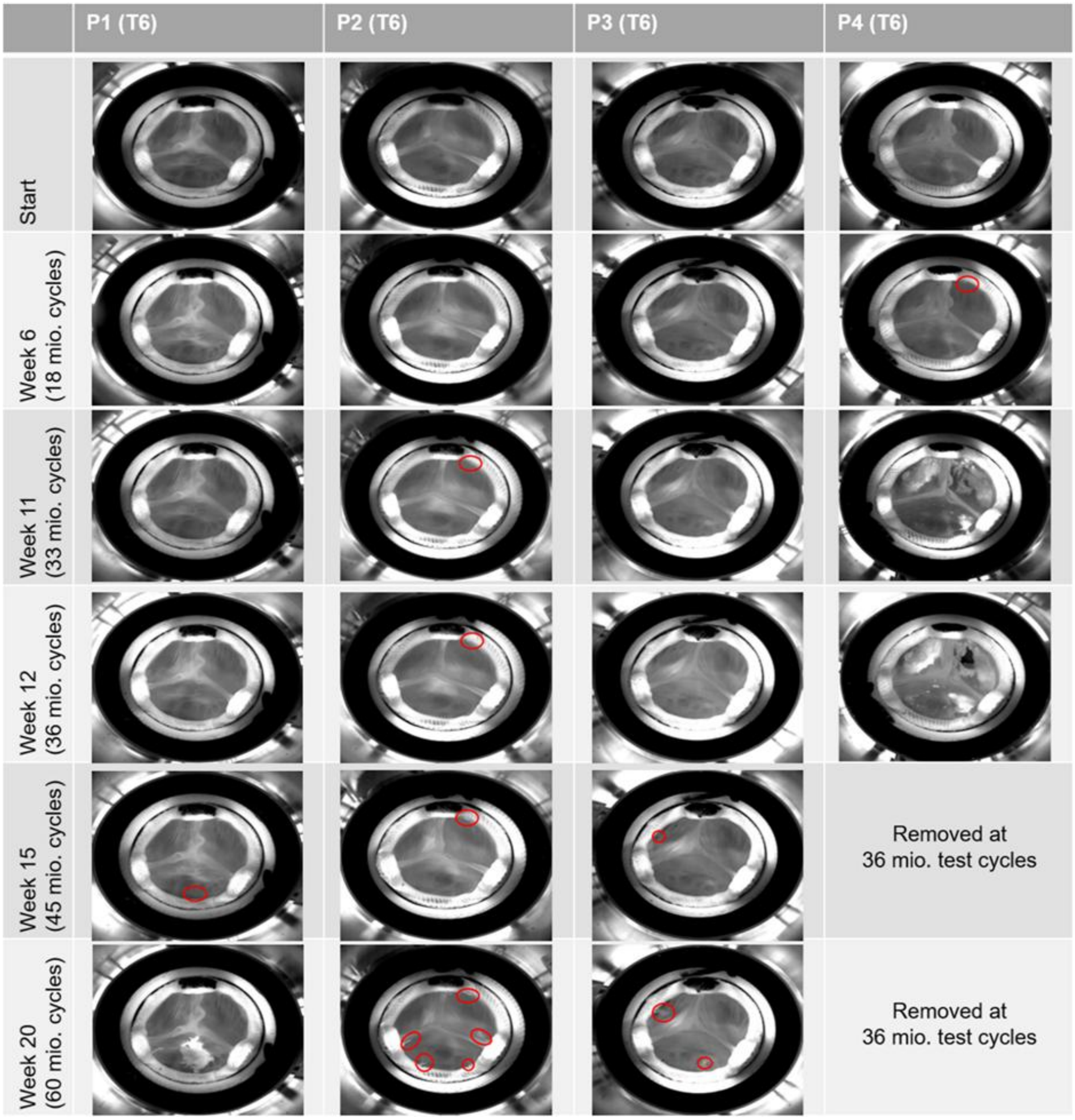

Fig. S1: HSV documentation of T6 treated prostheses for prominent test time points. Calcification onsets as well as initial increases are highlighted by red markings (for a better visualization of the calcification onsets and calcification progress at the end of the test, enlarged views are given in Fig. S1a – S1f)

Corresponding author: Johanna C. Clauser; clauser@ame.rwth-aachen.de

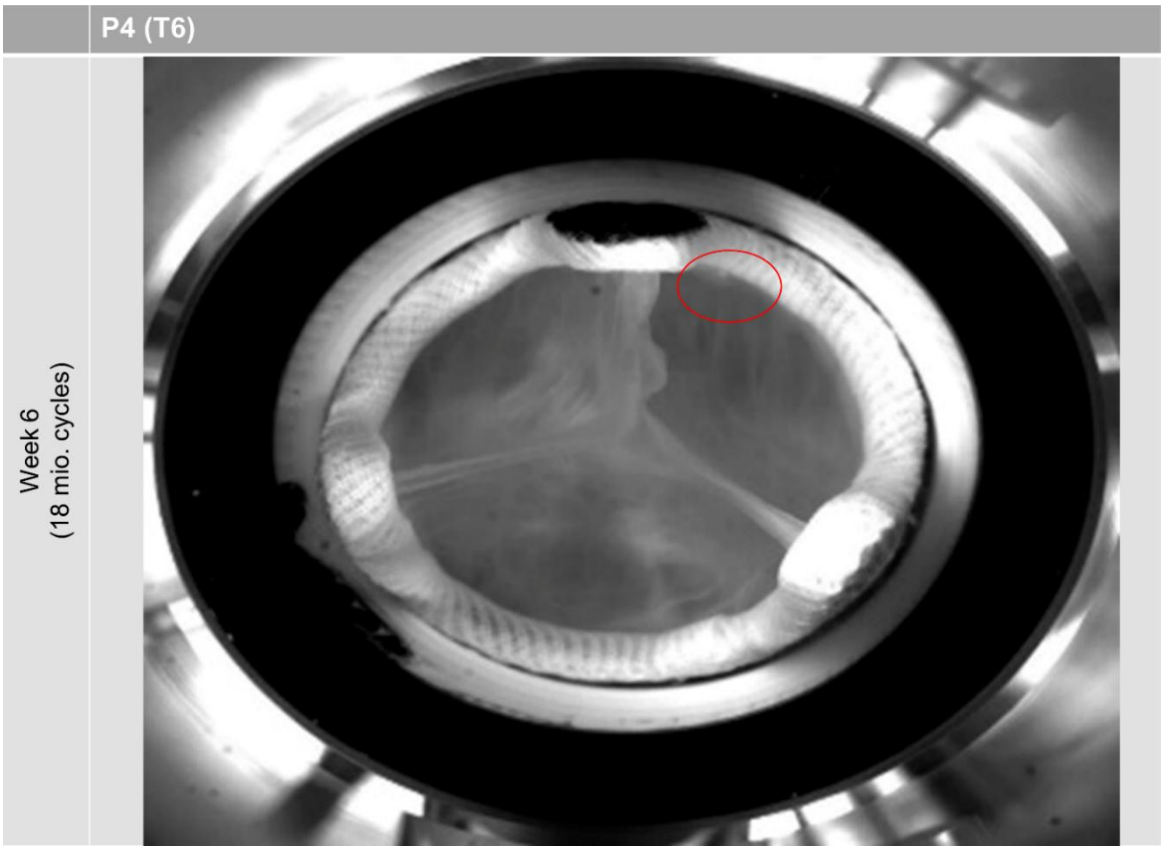

Fig. S1a: Calcification onset point of test prosthesis P4 (T6 group) by HSV detection

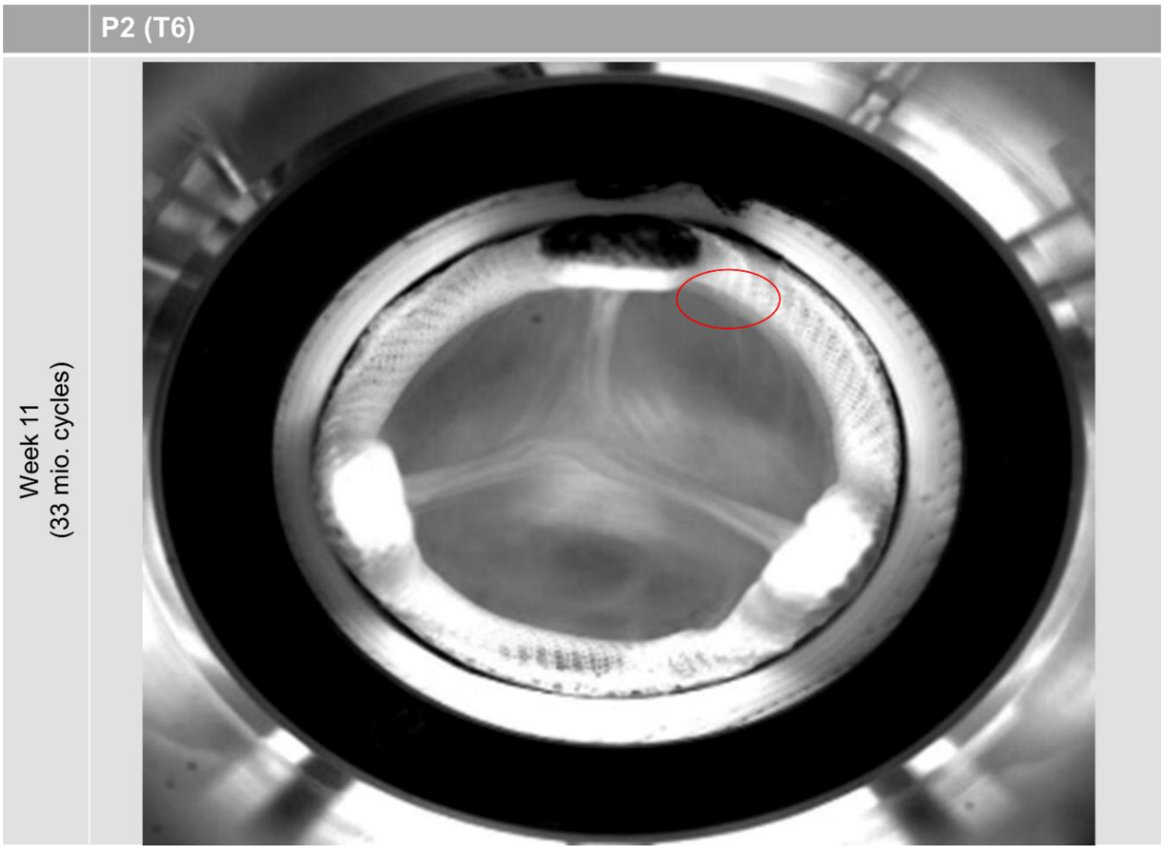

Fig. S1b Calcification onset point of test prosthesis P2 (T6 group) by HSV detection

Corresponding author: Johanna C. Clauser; clauser@ame.rwth-aachen.de

	P1 (T6)
Week 15 (45 mio. cycles)	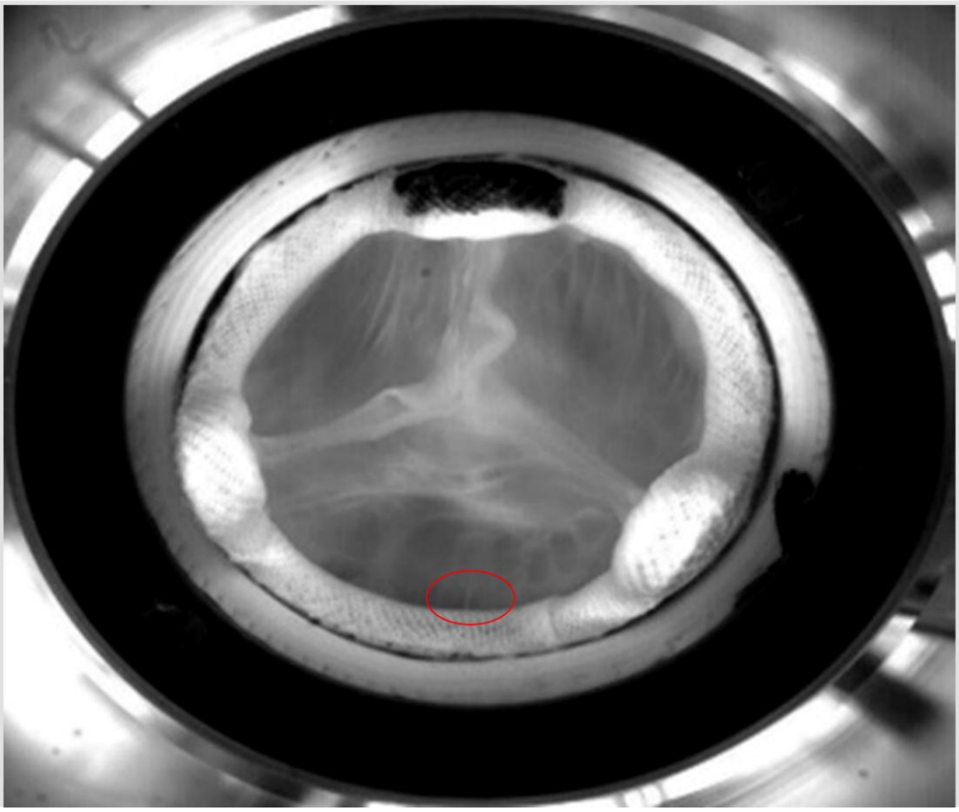

Fig. S1c Calcification onset point of test prosthesis P1 (T6 group) by HSV detection

	P3 (T6)
Week 15 (45 mio. cycles)	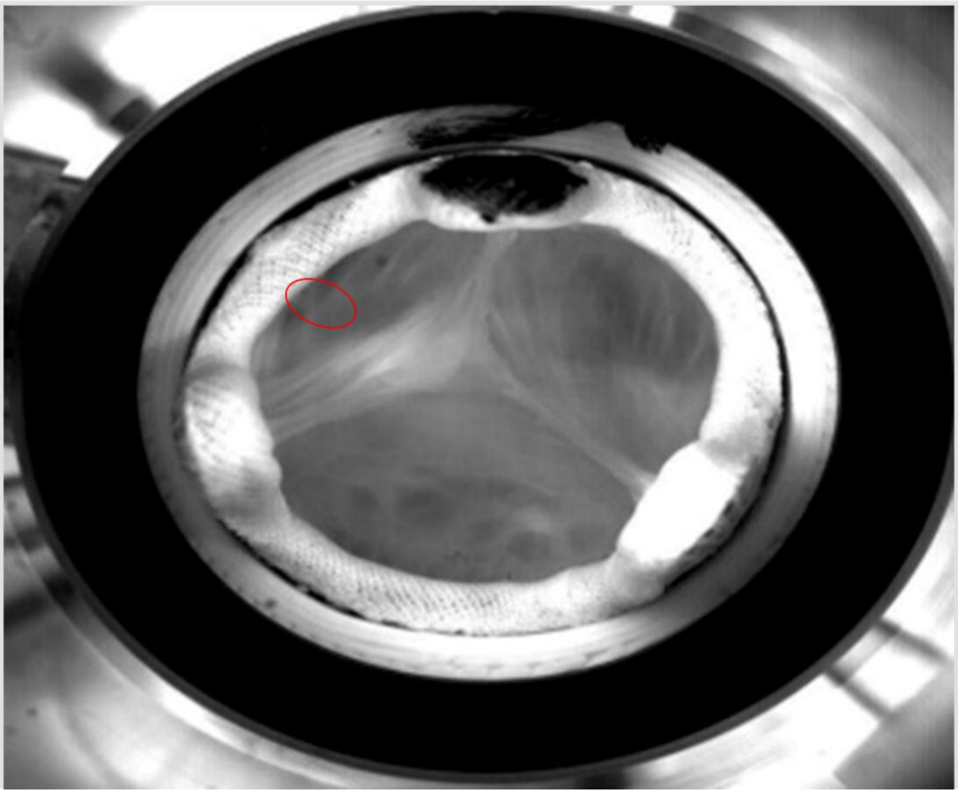

Fig. S1d Calcification onset point of test prosthesis P3 (T6 group) by HSV detection

Corresponding author: Johanna C. Clauser; clauser@ame.rwth-aachen.de

	P2 (T6)
Week 20 (60 mio. cycles)	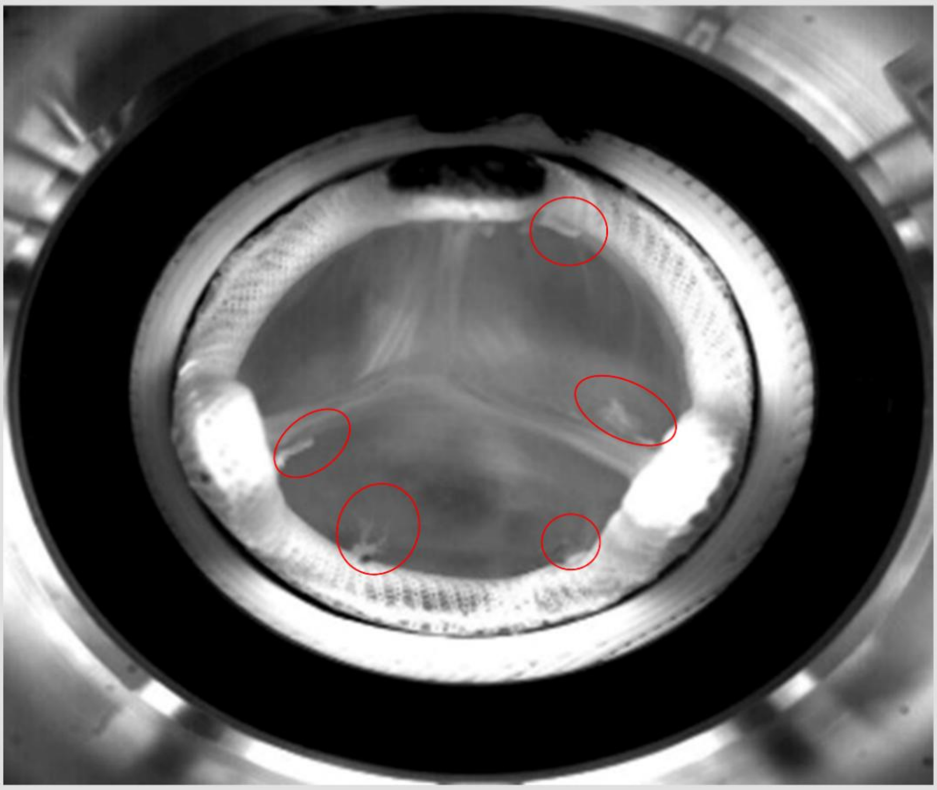

Fig. S1e Calcification progression of test prosthesis P2 (T6 group) at test end by HSV detection

	P3 (T6)
Week 20 (60 mio. cycles)	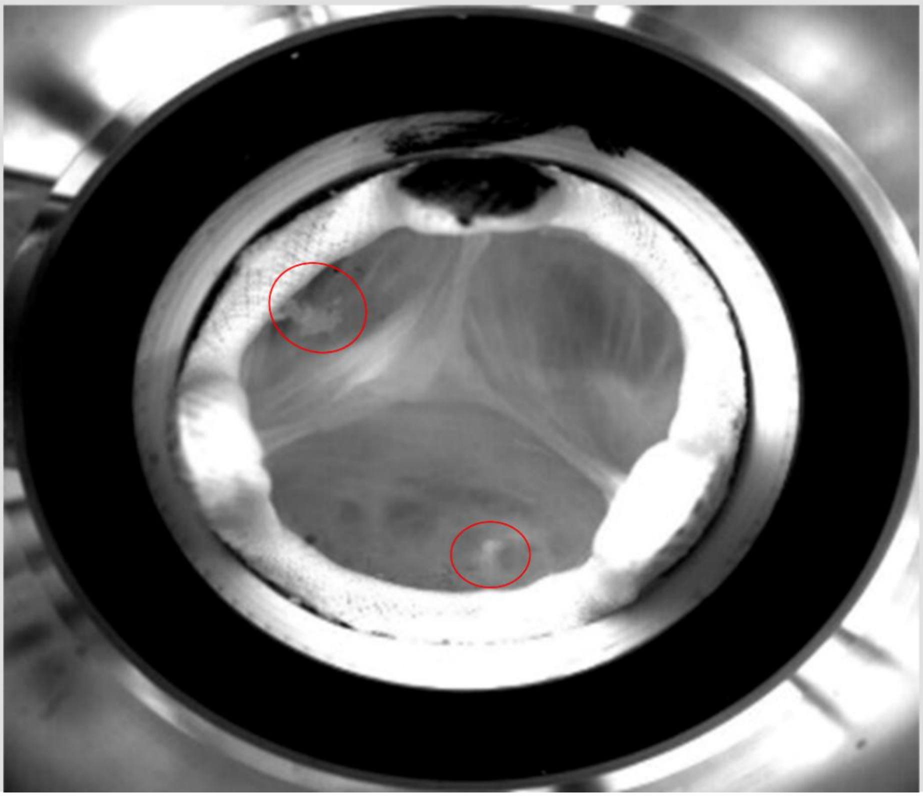

Fig. S1f Calcification progression of test prosthesis P2 (T6 group) at test end by HSV detection

Corresponding author: Johanna C. Clauser; clauser@ame.rwth-aachen.de

High-Speed Video Documentation – no-T6 treated prostheses

The very first visible signs of calcification were detected in the No-T6 group at 9 million test cycles (3 weeks) (Sample P6) (Fig. S2) and 15 million test cycles (5 weeks) (samples P5 and P7) (Fig. S2). Sample P8 showed the first sign of calcification at 24 million test cycles (8 weeks). However, the position of calcification onset for this prosthesis was so close under the suture ring that it could not be detected with the high-speed video camera, therefore an additional photograph of calcification onset is shown in Fig. S2. At the samples P6 and P7, increasing calcification within 9 to 10 weeks from the onset of calcification led to such leaflet stiffening followed by hole formation in the most calcified leaflet that these prostheses had to be removed from the test after 36 million (12 weeks) and 45 million test cycles (15 weeks), respectively (Fig. S2), because the required target peak differential pressure could not be adjusted anymore. In both prostheses, only the two larger leaflets were clearly affected by calcification until removal; the smaller leaflet showed only minor signs at most. At sample P5, only a slight increase in calcification was visible in the first 7 weeks after onset of calcification, and only after 39 million cycles (week 13) a distinct increase in calcification was observed, extending across all three leaflets at the end of testing (Fig. S2). Sample P8 also showed only a slight increase in calcification in the first 7 weeks after onset of calcification, which then increased massively from 45 million test cycles on (week 15). At the end of the test, this prosthesis revealed significant calcification of the two larger leaflets, but only slight calcification of the smaller leaflet (Fig. S2).

The onset points of the calcifications were predominantly found near the stent and suture ring areas during the high-speed video documentation (Fig. S1 and Fig. S2). When the prosthetic heart valves were later detached from the stent and suture ring for analytical purposes, suture insertion points were visible at these locations, which also indicates the influence of the mechanical stress occurring particularly at these attachment zones on the calcification process.

Corresponding author: Johanna C. Clauser; clauser@ame.rwth-aachen.de

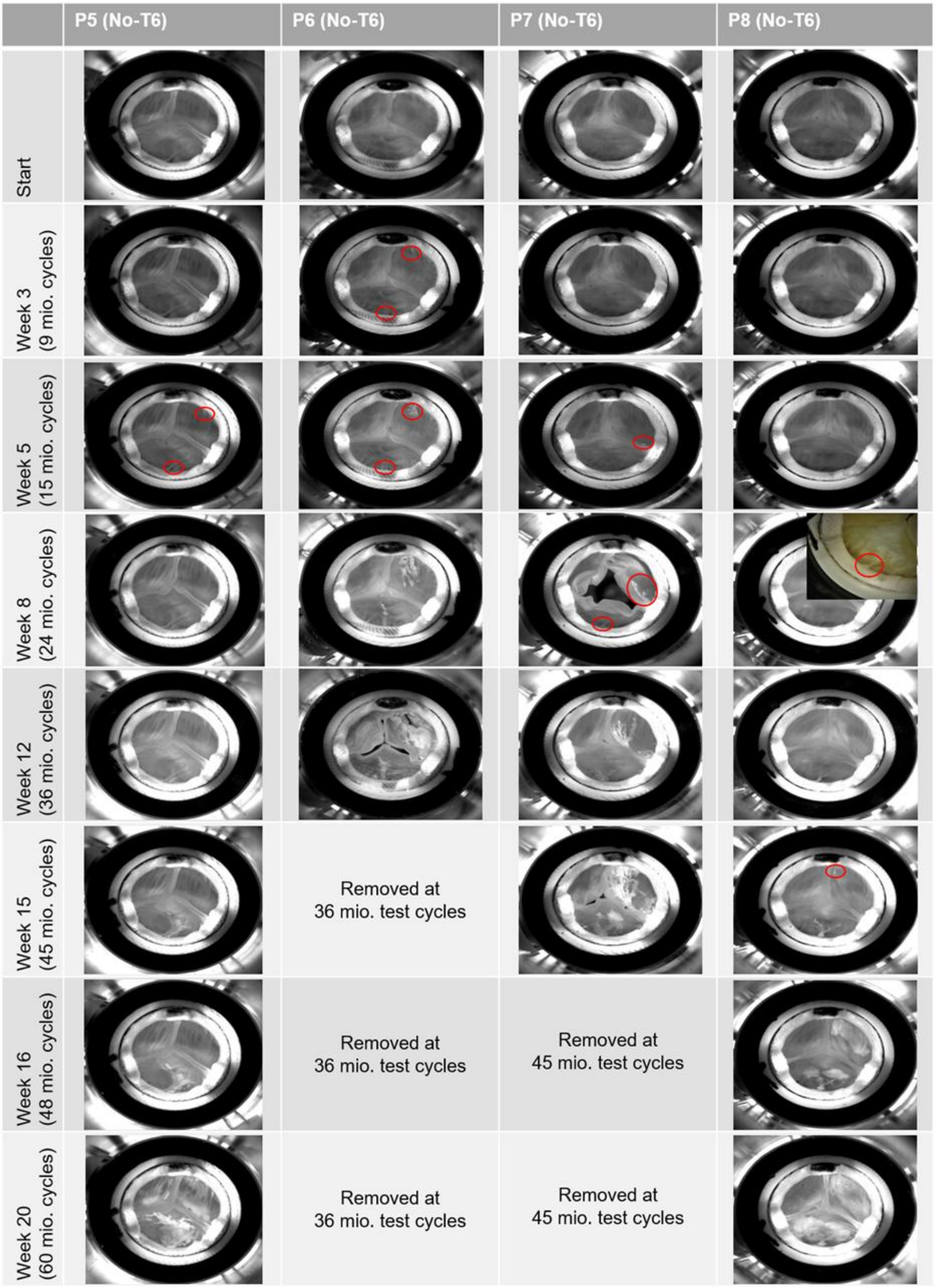

Fig. S2 HSV documentation of No-T6 treated prostheses for prominent test time points. Calcification onsets as well as initial increases are highlighted by red markings (for a better visualization of the calcification onsets, enlarged views are given in Fig. S2a – S2d)

Corresponding author: Johanna C. Clauser; clauser@ame.rwth-aachen.de

	P6 (No-T6)
Week 3 (9 mio. cycles)	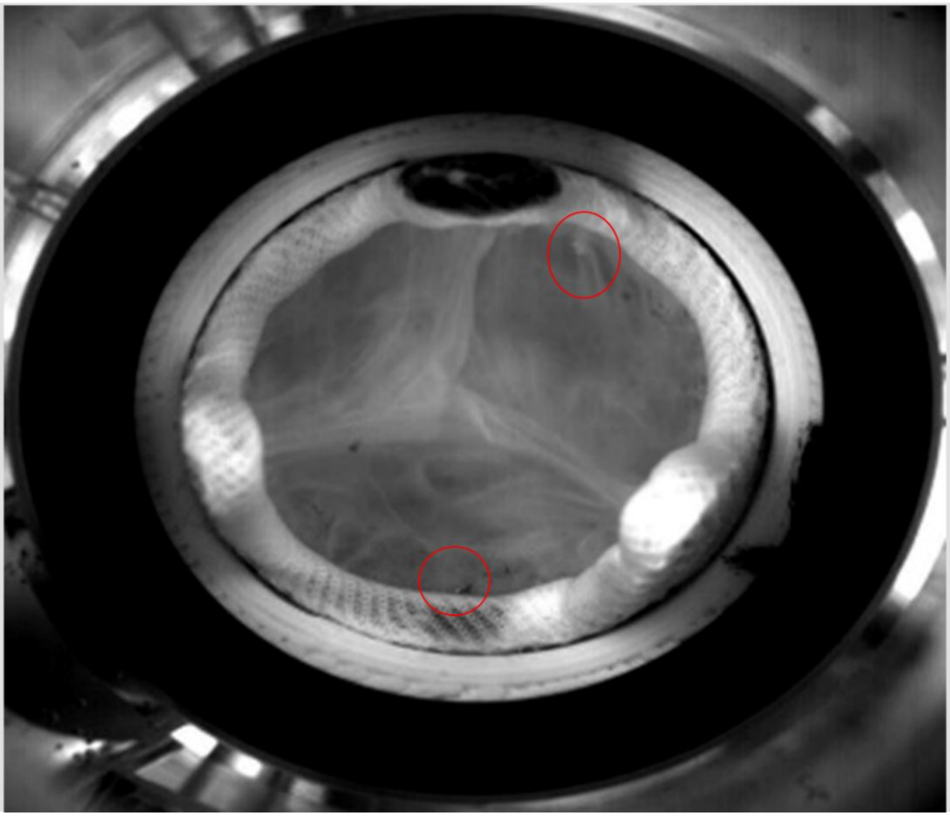

Fig. S2a Calcification onset points of test prosthesis P6 (No-T6 group) by HSV detection

	P5 (No-T6)
Week 5 (15 mio. cycles)	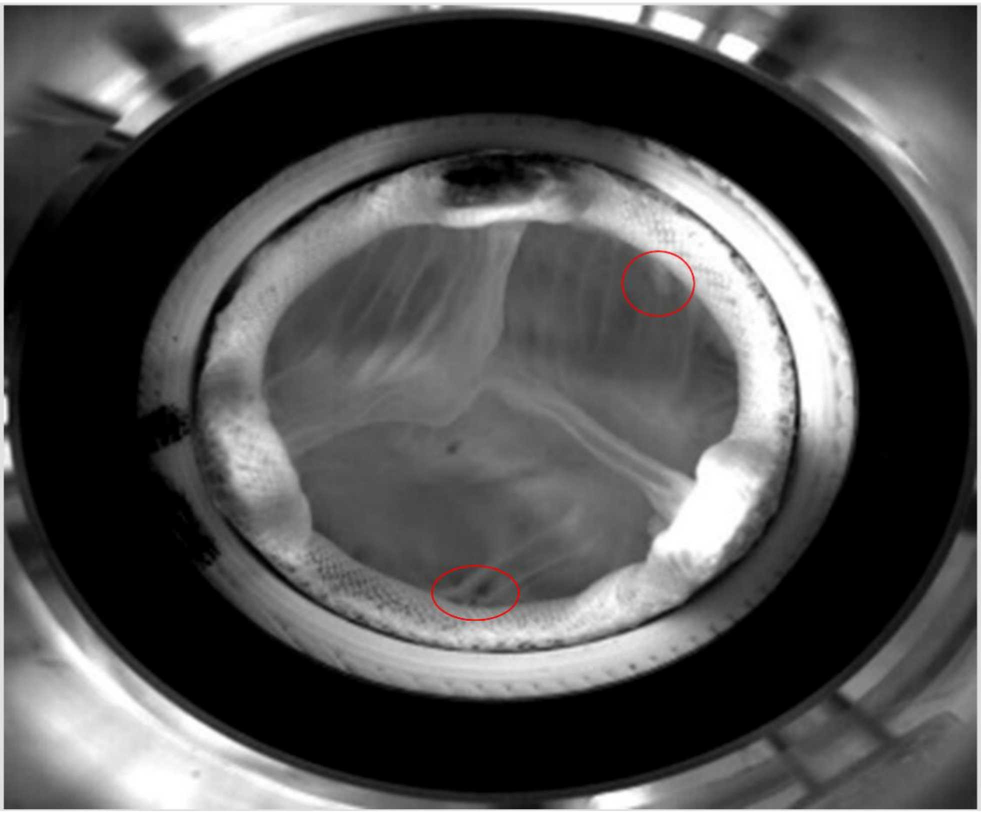

Fig. S2b Calcification onset points of test prosthesis P5 (No-T6 group) by HSV detection

Corresponding author: Johanna C. Clauser; clauser@ame.rwth-aachen.de

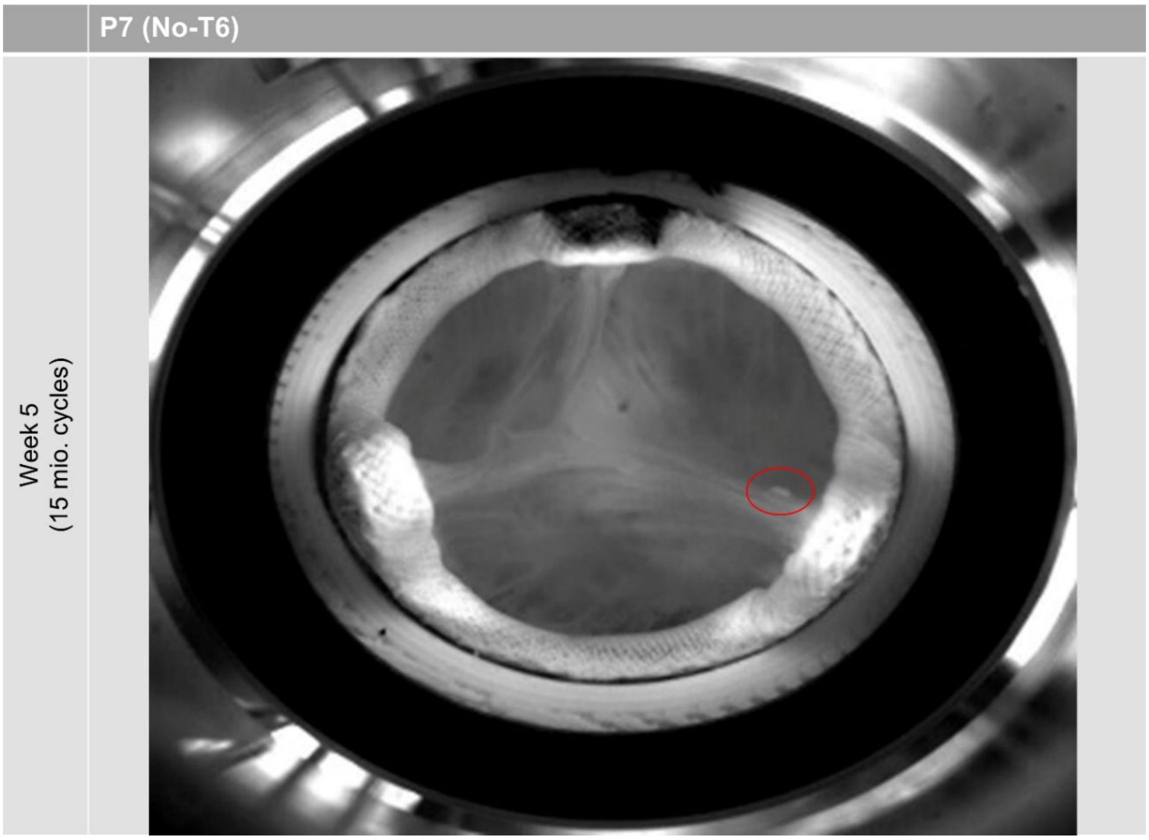

Fig. S2c Calcification onset points of test prosthesis P5 (No-T6 group) by HSV detection

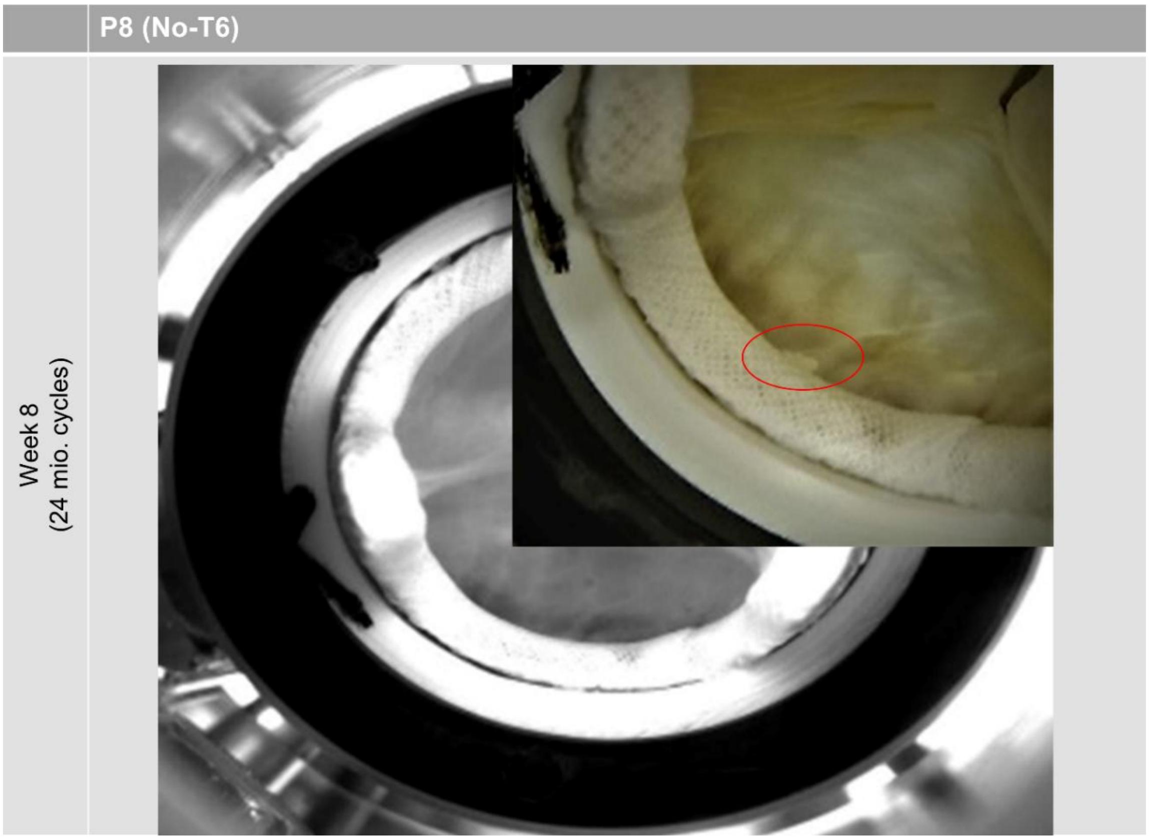

Fig. S2d Calcification onset points of test prosthesis P5 (No-T6 group) by HSV detection and additional photographic detection

Corresponding author: Johanna C. Clauser; clauser@ame.rwth-aachen.de

Chemical Quantification of Calcium and Phosphate by Colorimetry and Complexometry

Sample Preparation Process

For chemical quantification, systematically selected leaflet halves or the complete leaflets were used. The test prostheses separated from the stent were washed in distilled water before selection, in order not to carry over any residues of the storage solution into the analysis. The leaflet halves or whole leaflets collected per test prosthesis were dried in a desiccator under vacuum until mass constancy was achieved, and thus the dry weight of the respective mineralized valve tissue was determined. The dried tissue plus debris of each prosthesis (except for two strongly calcified prostheses) was immersed in hydrochloric acid 1N overnight and additionally in hydrochloric acid 6N for about one hour the next day to dissolve all mineralization out of the tissue. Subsequently, the tissue specimens per test prosthesis were rinsed several times with distilled water. The combined hydrochloric acid mineral extracts and the rinsing fractions from each test prosthesis were raised to pH~4 with sodium hydroxide solution 5N. Following this, they were quantitatively transferred into volumetric flasks (100 ml or 200 ml), depending on the expected amount of calcium (e.g. from µ-CT quantification) and filled up with distilled water. These solutions were applied for colorimetric and complexometric analysis.

Re-drying of the demineralized tissue pieces

The remaining demineralized tissue pieces were re-dried to constant weight to determine the tissue dry weight and additionally the mass of the deposits from the weight difference of mineralized and demineralized tissue. The leaflet halves of two strongly calcified test prostheses were placed directly in 6N hydrochloric acid overnight for mineral extraction due to the strong calcification according to µ-CT quantification. It became apparent that the tissue was also massively affected and disintegrated, so that reliable re-drying of the mineralization-free tissue of these two prostheses was no longer possible.

Colorimetric Analysis of Calcium and Phosphate

For colorimetric analyses of calcium and phosphate, the Vitros Chemistry System 350 Ortho Clinical Diagnostics (Vitros Ca method and Vitros Phos method) was used.

Approximately 1.5 mL were taken from each volumetric flask for calcium and phosphate determination, the actual sample volume was 10 µl / determination.

Based on the determined molar Ca / P ratios, an assignment of the calcifications formed to different calcium phosphate phases was made (**Table S1**). By means of their stoichiometry, the calcium masses determined per test material could be converted

Corresponding author: Johanna C. Clauser; clauser@ame.rwth-aachen.de

into the corresponding calcium phosphate masses. Since the phosphate value of test sample P3 was below the measuring range of the method and the calcium value was also in the lower limit range of the measuring method, no clear assignment could be made here. The assignment to the calcium phosphate phase OCP was made with reservations, since similar to the test samples P1 and P2, the calcification is still in the initial phase. The assignment to CDHA instead of OCP would not yield any considerable differences.

Table S1 Quantification of Calcium and Phosphate via colorimetric analysis and assignment to Calcium phosphate phases

Test prosthesis		Test material	Total volume of mineral extract	c (Ca) / mmol/l	c (P) / mmol/l	Molar Ca/P ratio	Calcium phophate phase	m (Ca) / total volume of mineral extract	m (Calcium phosphate phase) / test material
Standard9999999 T6 group	P1	3 leaflet halves	100 ml	0.43	0.32	1.34	OCP	1.72 mg	5.28 mg
	P2	3 whole leaflets	100 ml	1.13	0.73	1.55	CDHA	4.52 mg	11.72 mg
	P3	3 whole leaflets	100 ml	**0.14	under limit of measuring range	-	OCP under reserve	0.56 mg	1.72 mg
	P4	3 leaflet halves	200 ml	1.19	0.77	1.55	CDHA	9.52 mg	24.69 mg
No-T6 group	P5	3 leaflet halves	200 ml	0.99	0.69	1.43	CDHA	7.92 mg	20.54 mg
	P6	3 leaflet halves	200 ml	1.73	1.03	1.67	HAP	13.84 mg	34.76 mg
	P7	3 leaflet halves	200 ml	2.02	1.19	1.69	HAP	16.16 mg	40.59 mg
	P8	3 leaflet halves	200 ml	1.84	1.10	1.67	HAP	14.72 mg	36.97 mg

**lower limit of measuring range

For better comparison, the calcium masses determined per test material (3 leaflet halves or 3 leaflets) were normalized to mg Ca / g dry tissue (**Table S2**). When comparing the dry weight of the demineralized tissue obtained after subtracting the calculated calcium phosphate weight from the dry weight of the mineralized tissue with the dry weight obtained after re-drying the demineralized tissue, it appears that some valvular tissue is always lost during demineralization, especially for the tissue of the samples P7 and P8 (**Table S2**). For this reason, the tissue dry weight obtained after

Corresponding author: Johanna C. Clauser; clauser@ame.rwth-aachen.de

subtracting the weight of mineralization was chosen as the basis for normalization mg Ca / g tissue dry weight.

Table S2 Normalization of m (Ca) of colorimetric determination per g tissue dry weight

Test prosthesis		Test material	Dry weight of calcified test tissue	Dry weight of re-dried demineralized test tissue	Dry weight of test tissue after substraction of calculated calcification weight (from colorimetry)	m (Ca) / test material	m (Ca) / g tissue dry weight
Standard T6 group	P1	3 leaflet halves	0.1197 g	0.1086 g	0.1144 g	1.72 mg	15.03 mg
	P2	3 whole leaflets	0.2854 g	0.2586 g	0.2737 g	4.52 mg	16.51 mg
	P3	3 whole leaflets	0.2541 g	0.2417 g	0.2524 g	0.56 mg	2.22 mg
	P4	3 leaflet halves	0.1040 g	0.0760 g	0.0793 g	9.52 mg	120.05 mg
No-T6 group	P5	3 leaflet halves	0.1483 g	0.1225 g	0.1278 g	7.92 mg	61.97 mg
	P6	3 leaflet halves	0.1569 g	0.1132 g	0.1221 g	13.84 mg	113.35 mg
	P7	3 leaflet halves	0.1569 g	0.0047 g	0.1163 g	16.16 mg	138.95 mg
	P8	3 leaflet halves	0.1579 g	0.0036 g	0.1209 g	14.72 mg	121.75 mg

Complexometric Analysis and Calcium Determination

The complexometric calcium determination is a titrimetric method using ethylendiaminetetraacetic acid di-sodium salt (EDTA-Na_2) against indicator buffer tablets.

The calcium masses to be determined were obtained from the titration results (EDTA consumption) according to the following stoichiometric relationship (Eq. S1):

$$1 \text{ ml EDTA-Na}_2 \text{ (0.001M)} \equiv 0.04 \text{ mg } Ca^{2+} \quad \text{(Eq. S1)}$$

Corresponding author: Johanna C. Clauser; clauser@ame.rwth-aachen.de

For the complexometric calcium determination aliquots of the calcium-containing test solutions were titrated with 0.001M EDTA at pH 10 - 11 (adjusted with ammonia solution) against indicator buffer tablets until a color change from red to green occurs. Each determination was performed as triple determination. Distilled water was determined as a blank sample.

The calcium values obtained per aliquot were extrapolated to the respective total volume of the mineral extracts (**Table S3**). The calcifications formed were assigned to calcium phosphate phases on the basis of the calcium values determined by complexometry (**Table S3**) and phosphate values determined by colorimetry (**Table S1**). Except for test sample P1, there was no relevant deviation from the colorimetrically determined Ca / P ratios.

Table S3 Quantification of calcium via complexometric titration

Test prosthesis		Test material	Total volume of mineral extrakt	aliquot test volume	V (EDTA-Na_2 0.001M)	m (Ca^{2+}) / aliqot test volume	m (Ca^{2+}) / total volume of mineral extract	c (Ca) / mmol/l	Calcium phos-phate phase	m (Calcium phos-phate phase) / test material
Standard T6 group	P1	3 leaflet halves	100 ml	20 ml	10.10 ml	0.404 mg	2.02 mg	0.51	CDHA	5.24 mg
	P2	3 whole leaflets	100 ml	10 ml	11.80 ml	0.472 mg	4.72 mg	1.18	CDHA	12.24 mg
	P3	3 whole leaflets	100 ml	20 ml	3.35 ml	0.134 mg	0.67 mg	0.17	OCP under reserve	2.06 mg
	P4	3 leaflet halves	200 ml	10 ml	12.25 ml	0.490 mg	9.80 mg	1.23	CDHA	25.41 mg
No-T6 group	P5	3 leaflet halves	200 ml	10 ml	10.60 ml	0.424 mg	8.48 mg	1.06	CDHA	21.99 mg
	P6	3 leaflet halves	200 ml	10 ml	16.70 ml	0.668 mg	13.36 mg	1.67	HAP	33.55 mg
	P7	3 leaflet halves	200 ml	10 ml	19.60 ml	0.784 mg	15.68 mg	1.96	HAP	39.38 mg
	P8	3 leaflet halves	200 ml	10 ml	18.70 ml	0.748 mg	14.96 mg	1.87	HAP	37.57 mg

For better comparison, the calcium masses determined per test material (3 leaflet halves or 3 leaflets) were normalized to mg Ca / g dry tissue (**Table S4**).

Corresponding author: Johanna C. Clauser; clauser@ame.rwth-aachen.de

Table S4 Normalization of m (Ca) of complexometric determination per g tissue dry weight

Test prosthesis		Test material	Dry weight of calcified test tissue	Dry weight of re-dried demineralized test tissue	Dry weight of test tissue after substraction of calculated calcification weight (from comlexometry)	m (Ca) / test material	m (Ca) / g tissue dry weight
Standard T6 group	P1	3 leaflet halves	0.1197 g	0.1086 g	0.1145 g	2.02 mg	17.64 mg
	P2	3 whole leaflets	0.2854 g	0.2586 g	0.2732 g	4.72 mg	17.28 mg
	P3	3 whole leaflets	0.2541 g	0.2417 g	0.2520 g	0.67 mg	2.66 mg
	P4	3 leaflet halves	0.1040 g	0.0760 g	0.0786 g	9.80 mg	124.68 mg
No-T6 group	P5	3 leaflet halves	0.1483 g	0.1225 g	0.1263 g	8.48 mg	67.14 mg
	P6	3 leaflet halves	0.1569 g	0.1132 g	0.1233 g	13.36 mg	108.35 mg
	P7	3 leaflet halves	0.1569 g	0.0047 g	0.1175 g	15.68 mg	133.45 mg
	P8	3 leaflet halves	0.1579 g	0.0036 g	0.1203 g	14.96 mg	124.36 mg

Comparison of the Different Quantification Methods

A direct numerical comparison of the quantifications via µ-CT and the two chemical methods (**Fehler! Verweisquelle konnte nicht gefunden werden.**) cannot be made, as µ-CT considers the calcifications present on the whole prostheses, whereas the chemical methods are based on a conversion of the calcification of representative leaflet sections to 1 g of dry tissue. Furthermore, it should be noted that the quantification via the µ-CT method is calculated for HAP as the only calcium phosphate phase formed (due to the limited calibration possibility).

Corresponding author: Johanna C. Clauser; clauser@ame.rwth-aachen.de

Structural Investigation of in vitro formed Calcifications via XRD

In pathological in-vivo calcification of both native and bioprosthetic heart valves, octacalcium phosphate (OCP) and hydroxyapatite (HAP) or a mixture of both occur as predominant phases [5, 6, 9, 15, 17]. Hydroxyapatite is the thermodynamically most stable and final phase. OCP, on the other hand, is considered to be a transitional phase which, in the course of time, transforms into the more stable HAP, depending on Ca^{2+} availability [17, 18].

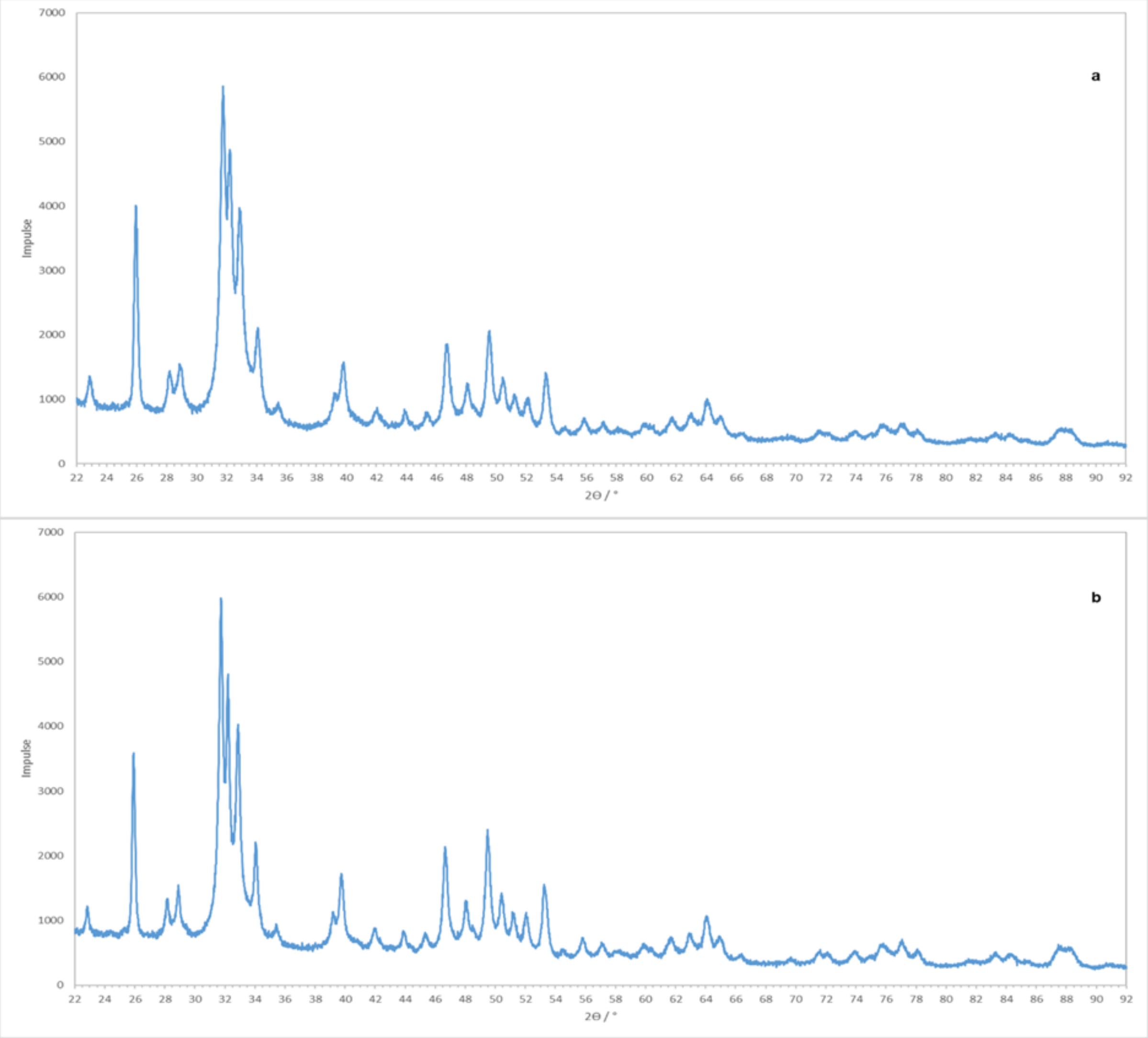

Fig. S3 X-ray powder diffractograms of deposits from prosthesis P4 (a) and prosthesis P5 (b)

The diffractograms were analyzed by means of structural refinement using the Rietveld-method [10] within the software suite Topas Academic 7 (Coelho Software, Australia). The apatite from P4 has lattice parameters *a* = 9.453(7) Å and *c* = 6.881(6) Å, the apatite from P5 has lattice parameters *a* = 9.447(7) Å and *c* = 6.879(6) Å in space group $P6_3/m$ [9]. These values are virtually identical for both samples within error bars.

Corresponding author: Johanna C. Clauser; clauser@ame.rwth-aachen.de

Histological Examination

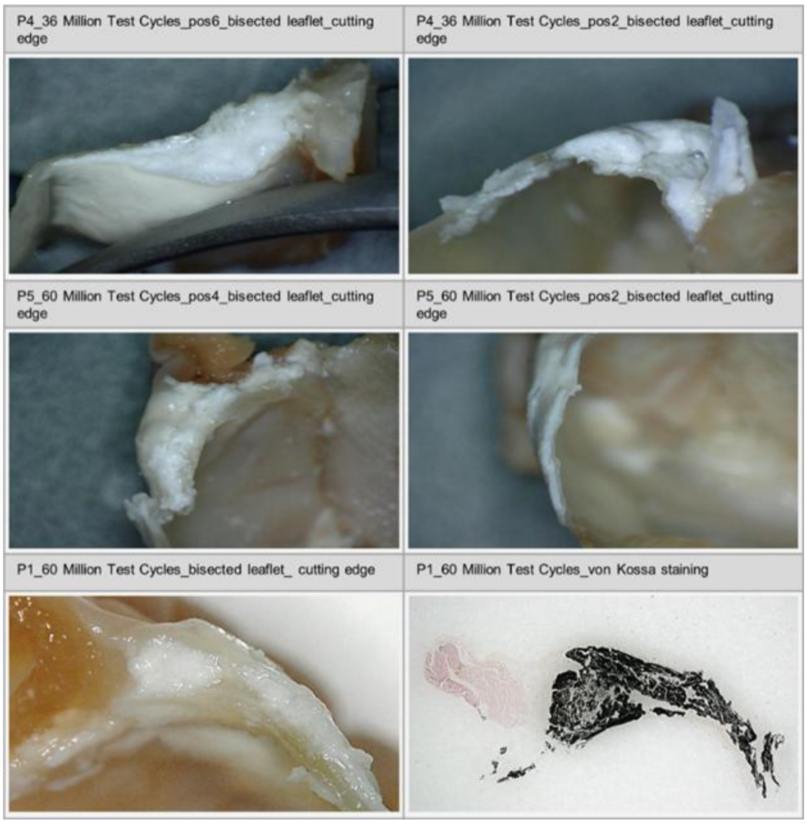

Fig. S4 Cutting edges of calcified bisected leaflets of the test samples P4 and P5 and comparison of the leaflet cutting edge and von Kossa staining of the calcified leaflet of test sample P1

A further interesting aspect of the calcification test method becomes apparent in prosthesis P4. It seems to have an exceptional position in the standard T6 group according to HSV recording and microscopic documentation as well as all three analysis methods. This may be due to natural tissue anomalies in biological material, which are not eliminated by fixation and pre-treatment and cannot be visually assessed in advance, but which become apparent in the calcification test method.

Corresponding author: Johanna C. Clauser; clauser@ame.rwth-aachen.de

Statistical comparison of the two test groups concerning their quantitative tendency to calcify

Table S5 Statistical evaluation of group differences with respect to mean HAP masses / prosthesis (μ-CT)

Test group	N	mg HAP / prosthesis mean ± S.E.M. (standard error of the mean)	Significance
Hancock II Ultra T505 (T6) (P1 - P4)	4	31.21 ± 17.45	$p < 0.05$
Hancock II Ultra T505 (No-T6) (P5 – P8)	4	86.07 ± 7.39	

Table S6 Statistical evaluation of group differences with respect to mean Ca masses / prosthesis (μ-CT)

Test group	N	mg Ca / prosthesis mean ± S.E.M.	Significance
Hancock II Ultra T505 (T6) (P1 - P4)	4	12.43 ± 6.95	$p < 0.05$
Hancock II Ultra T505 (No-T6) (P5 – P8)	4	34.77 ± 2.46	

Table S7 Statistical evaluation of group differences with respect to mean Ca masses / g dry tissue weight (colorimetry)

Test group	N	mg Ca / g tissue dry weight mean ± S.E.M.	Significance
Hancock II Ultra T505 (T6) (P1 - P4)	4	38.45 ± 27.39	$p = 0.070$
Hancock II Ultra T505 (No-T6) (P5 – P8)	4	109.01 ± 16.56	

Table S8 Statistical evaluation of group differences with respect to mean Ca masses / g dry tissue weight (complexometry)

Test group	N	mg Ca / g tissue dry weight mean ± S.E.M.	Significance
Hancock II Ultra T505 (T6) (P1 - P4)	4	40.57 ± 28.25	$p = 0.077$
Hancock II Ultra T505 (No-T6) (P5 – P8)	4	108.33 ± 14.68	

Corresponding author: Johanna C. Clauser; clauser@ame.rwth-aachen.de

Statistical comparison of the two test groups concerning their quantitative tendency to calcify

Table S9 Statistical evaluation of group differences with respect to mean Ca masses / g dry tissue weight, adapted from Jones et al. [8]

Valve Type	N	mg Ca / g tissue dry weight mean ± S.E.M.	Significance
Hancock II porcine aortic valves (T6)	17	17.7 ± 5.3	$p < 0.001$
Standard Hancock porcine aortic valves (No-T6)	28	64.7 ± 9.6	

Corresponding author: Johanna C. Clauser; clauser@ame.rwth-aachen.de
